# T78. LONG-TERM PROGNOSIS OF SCHIZOPHRENIA - RESULTS FROM THE NORTHERN FINLAND BIRTH COHORT 1966

**DOI:** 10.1093/schbul/sby016.354

**Published:** 2018-04-01

**Authors:** Erika Jääskeläinen, Matti Penttilä, Jani Moilanen, Marianne Haapea, Anja Hulkko, Irina Rannikko, Nina Rautio, Tanja Nordström, Juha Käkelä, Annika Seppälä, Johanna Immonen, Hanna Korpela, Jussi Seppälä, Graham Murray, Hannu Koponen, Matti Isohanni, Jouko Miettunen

**Affiliations:** 1 University of Oulu; 2 University of Cambridge; 3 University of Helsinki, Helsinki University Hospital

## Abstract

**Background:**

The aim of this study was to explore the prognosis and predictors of outcomes in schizophrenia in a birth cohort sample followed since mid-pregnancy until the age of 45 years.

**Methods:**

The sample included subjects with schizophrenia (n=29–161, depending on the analyzed topic) from the Northern Finland Birth Cohort 1966. Outcomes and their predictors were analyzed by utilizing national registers, questionnaires and personal examinations made on several time points (e.g. during pregnancy, at age 1 year, 34- and 43- years). Functioning, amount of psychiatric symptoms, utilization of treatments, physical illnesses and mortality, and cognition were used as measures of outcomes. Several plausible factors associating to outcomes were studied, e.g. gender, family history of psychosis, development and childhood related factors, school performance, and illness related factors around the onset of psychosis, brain morphology and cognitive functioning, and lifetime antipsychotic medication.

**Results:**

Around the age of 34-years recovery was possible though quite uncommon (3.4%), some persons achieved symptomatic remission (21%), and many were on disability pension (54%). Around the age of 43–45 years only 11.2% were employed, and 19% were in remission. Earlier age of illness onset, longer duration of untreated psychosis, suicidal ideation and poorer functioning around illness onset, brain morphological changes and poorer cognition, and higher lifetime doses of antipsychotics associated to poor outcomes. Cognition did not markedly decline from 34 to 43 years of age, but poorer premorbid school performance and higher lifetime doses of antipsychotics predicted more decline of cognition. For some cases, the cumulative amount of used antipsychotics was extensive. Somatic comorbidities were common, and mortality high.

**Discussion:**

Based on this naturalistic sample, progression of schizophrenia may follow a variety of different trajectories. Poor clinical course is common but not necessary outcome. Our results indicate heterogeneous and still relatively unsatisfactory prognosis of schizophrenia in this sample. Several predictors of outcomes have been found, and especially factors related to illness onset and high lifetime cumulative dose of antipsychotics are of interest. Birth cohort setting offers unique possibility to study long-term prognosis of schizophrenia.

